# Hypocalcemia development in patients operated for primary hyperparathyroidism: Can it be predicted preoperatively?

**DOI:** 10.1590/2359-3997000000207

**Published:** 2016-09-26

**Authors:** Cafer Kaya, Abbas Ali Tam, Ahmet Dirikoç, Aylin Kılıçyazgan, Mehmet Kılıç, Şeyda Türkölmez, Reyhan Ersoy, Bekir Çakır

**Affiliations:** 1 Ataturk Training and Research Hospital Department of Endocrinology and Metabolism Ankara Turkey Ataturk Training and Research Hospital, Department of Endocrinology and Metabolism, Ankara, Turkey; 2 Yıldırım Beyazıt University Department of Pathology Ankara Turkey Yıldırım Beyazıt University, Department of Pathology, Ankara, Turkey; 3 Yıldırım Beyazıt University Department of General Surgery Ankara Turkey Yıldırım Beyazıt University, Department of General Surgery, Ankara, Turkey; 4 Ataturk Training and Research Hospita Department of Nuclear Medicine Ankara Turkey Ataturk Training and Research Hospital, Department of Nuclear Medicine, Ankara, Turkey; 5 Yıldırım Beyazıt University Department of Endocrinology and Metabolism Ankara Turkey Yıldırım Beyazıt University, Department of Endocrinology and Metabolism, Ankara, Turkey

**Keywords:** Primary hyperparathyroidism, postoperative hypocalcemia, hungry bone syndrome

## Abstract

**Objective:**

Primary hyperparathyroidism (PHP) is a common endocrine disease, and its most effective treatment is surgery. Postoperative hypocalcemia is a morbidity of parathyroid surgeries, and it may extend hospitalization durations. The purpose of this study is to determine the predictive factors related to the development of hypocalcemia and hungry bone syndrome (HBS) in patients who underwent parathyroidectomy for PHP.

**Materials and methods:**

Laboratory data comprising parathyroid hormone (PTH), calcium, phosphate, 25-OHD, albumin, magnesium, alkaline phosphatase (ALP), blood urea nitrogen (BUN), and thyroid stimulating hormone (TSH) of the patients were recorded preoperatively, on the 1^st^ and 4^th^ days postoperatively, and in the 6^th^ postoperative month, and their neck ultrasound (US) and bone densitometry data were also recorded.

**Results:**

Hypocalcemia was seen in 63 patients (38.4%) on the 1^st^ day after parathyroidectomy. Ten patients (6.1%) had permanent hypocalcemia in the 6^th^ month after surgery. Out of the patients who underwent parathyroidectomy for PHP, 22 (13.4%) had HBS. The incidence of postoperative hypocalcemia was higher in patients who underwent parathyroidectomy for PHP, who had parathyroid hyperplasia, and who had osteoporosis. Preoperative PTH, ALP, and BUN values were higher in those patients who developed HBS. Furthermore, HBS was more common in patients who had osteoporosis, who had parathyroid hyperplasia, and who underwent thyroidectomy simultaneously with parathyroidectomy.

**Conclusions:**

As a result, patients who have the risk factors for development of hypocalcemia and HBS should be monitored more attentively during the perioperative period.

## INTRODUCTION

Primary hyperparathyroidism is a common endocrine disease, and more than 80% of patients have solitary adenomas, which cause long-term and excess secretion of parathyroid hormone (PTH) (1,2). Its prevalence increases with old age, and it is more common in women (3). Parathyroidectomy is the only definitive treatment that can normalize calcium and PTH levels (4). Hypocalcemia is a common complication that occurs after parathyroidectomy (5,6). Temporary postoperative hypocalcemia has been reported to have a prevalence of 0-35%, while permanent hypocalcemia has been reported to have a prevalence of 0-3.8% in the medical literature (7). Carpopedal spasms, perioral paresthesia, tingling extremities, Chvostek sign and Trousseau sign can be seen as associated with neuromuscular irritability (8). While it usually exhibits minor symptoms, less frequently, it may also cause cardiac arrhythmias, myocardial dysfunction, and pulmonary edemas (5).

The reasons for development of postoperative hypocalcemia are multifactorial. It is associated with excision or biopsy of more than two parathyroid glands, accompanying thyroid surgery, history of previous neck surgery, inadvertent gland removal, hemodilution, temporary vascular compromise of the remaining parathyroid tissue, and long-term hypocalcemia suppression of non-adenomatous parathyroid tissue (3,9,10). One of the reasons for postoperative hypercalcemic is hungry bone syndrome (HBS). This syndrome may occur in patients who develop increased bone resorption during the preoperative phase. PTH stimulus is abruptly removed after parathyroidectomy, and hence, excess osteoclastic activity stops, but osteoblastic activity continues, and excess calcium and phosphorus pass into the bones (8).

Many experienced physicians recommend 1 to 3 days of hospital stay for the patient’s safety due to fear of untreated hypocalcemia and tetany (11). Airway obstruction and recurrent nerve damage are rare. Postoperative hypocalcemia is the only reason that physicians keep patients in the hospital for more than 24 hours after surgery (3). The purpose of this study is to establish the predictive factors in patients who develop postoperative hypocalcemia and HBS after they undergo parathyroidectomy for primary hyperparathyroidism.

## MATERIALS AND METHODS

One hundred and sixty-four patients, who underwent parathyroidectomy for primary hyperparathyroidism at the Ankara Ataturk Training and Research Hospital between 2004 and 2014, were included in the study. Those who had secondary and tertiary hyperparathyroidism, and who had relapses and recurrences, were not included in the study. Laboratory data comprising PTH, calcium, phosphate, 25-OHD, albumin, magnesium, alkaline phosphatase (ALP), blood urea nitrogen (BUN), and thyroid stimulating hormone (TSH) of the patients were recorded preoperatively on the 1^st^ and 4^th^ days postoperatively and in the 6^th^ postoperative month, and their neck ultrasounds and bone densitometry data were also recorded. The normal ranges of the laboratory parameters were established as follows: PTH: 15 – 65 pg/mL, Ca: 8.6 – 10 mg/dL, phosphorus: 2.5 – 4.5 mg/dL, albumin: 3.5 – 5.2 g/dL, magnesium: 1.6 – 2.6 mg/dL, ALP: 34 – 105 IU/L, BUN: 10 – 48.5 mg/dL, TSH: 0.27 – 4.2 IU/mL, fT3: 2 – 4.4 pg/mL, fT4: 0.9 – 1.7 ng/dL, 25-OHD: 30 – 100 ng/mL. There is a passionate discussion about the normal range of the serum levels of 25-OHD. We accepted the normal range of values accepted by the Endocrine Society. In our study, while vitamin D deficiency was considered to be lower than 20 ng/mL, insufficiency was considered to be in the range of 21–29 ng/mL. Their calcium levels were calculated with the corrected calcium formula based on their albumin levels before and after parathyroidectomy (Corrected Ca (mg/dL) = [0.8 × (normal albumin – patient’s albumin)] + serum Ca level). Postoperative serum calcium levels below the normal range were defined as biochemical hypocalcemia (≤ 8.5 mg/dL).

The patients who had hypocalcemia and hypophosphatemia (≤ 8.5 mg/dL and < 2.5 mg/dL, respectively) on the 4^th^ postoperative day were considered to have HBS. All blood samples were collected after overnight fasting. Parathyroid volume was calculated using the ellipsoid model formula (length × width × height × 0.52). Four-gland parathyroid explorations were performed on all the patients. The surgeries were performed by the same surgical unit. The same surgical procedure was performed on those who had parathyroid hyperplasia. None of the cases had recurrence throughout their follow-ups. No parathyroid carcinomas were encountered in our set of patients. Thyroidectomy was performed on patients for multinodular goiter, Graves’ disease, toxic multinodular goiter, and goiter with compression symptoms.

PTH, TSH, fT3, and fT4 were measured using Roche Cobas Elecsys 601, and Ca, phosphorus, albumin, magnesium, alkaline phosphatase, and BUN were measured using Roche Cobas colorimetric 501, and 25-OHD was measured using LC-MS Shimadzu API 3200.

This study was approved by the Research Ethics Committee of the institution under protocol No. 34/2014. The approval of the medical ethics committee was obtained. The requirement for patient informed consent was waived.

The data obtained from this study were analyzed using the SPSS 20 package program. In addition to the frequency and percentage representations of the data, Chi-square dependency test was used for assessment of association of categorical data, and Student’s t-test and Mann-Whitney U-test were used for two-group comparisons. Significance level was taken as 0.05, p < 0.05 was considered an existence of a significant difference, and p > 0.05 was considered an absence of a significant difference.

## RESULTS

Out of the 164 patients included in the study, 23 (14%) were male, and 141 (86%) were female. Hypocalcemia was seen in 63 patients (38.4%) on the 1^st^ day after parathyroidectomy. Ten patients (6.1%) had permanent hypocalcemia in the 6^th^ month after surgery. While no significant difference was found with regards to demographics, early hypocalcemia was more common in females (p < 0.05). Solitary adenomas were identified in 147 operations, double adenomas were identified in 7 operations, and 10 patients had hyperplasia. While there was no significant difference with regard to parathyroid volume and development of early hypocalcemia, parathyroid volumes of the patients who developed permanent hypocalcemia were significantly higher (p < 0.05). Hypocalcemia was more common in patients with parathyroid hyperplasia (80%, p < 0.05).

Simultaneous thyroid operations were performed on 89 patients (54.3%): 21 of them were subtotal, and 68 were total thyroidectomies. Thyroid carcinoma was detected in 3 of the patients who underwent thyroid surgery (3/89, 3.4%). All 3 cases were papillary thyroid carcinoma, and all were treated with radioactive iodine. Early hypocalcemia was more common in patients who underwent total thyroidectomy compared to those who underwent subtotal thyroidectomy and those who did not undergo thyroid surgery (p < 0.05). Statistical evaluation could not be performed for concomitant thyroid surgery with regards to permanent hypocalcemia because of the small number of patients. This situation did not affect the development of hypocalcemia in patients who had a history of neck surgery (n = 7) either.

There was no statistically significant difference between preoperative PTH, calcium, phosphorus, magnesium, albumin, BUN, TSH, and vitamin D levels and development of early and permanent hypocalcemia (Tables 1 and 2). Nineteen of the patients who developed early hypocalcemia had osteoporosis, and 30 of them had osteopenia. Early hypocalcemia was more common in patients with osteoporosis compared to those with normal BMD (bone mineral density) and those with osteopenia (p < 0.05).

**Table 1:**
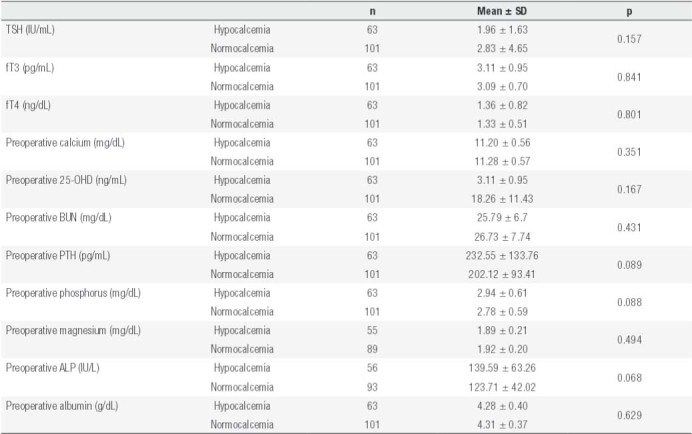
Laboratory characteristics of patients who developed hypocalcemia on postoperative day 1

Normocalcemia was determined as between 8.6 and 10.4 and hypocalcemia as ≤ 8.5 mg/dL.

**Table 2:**
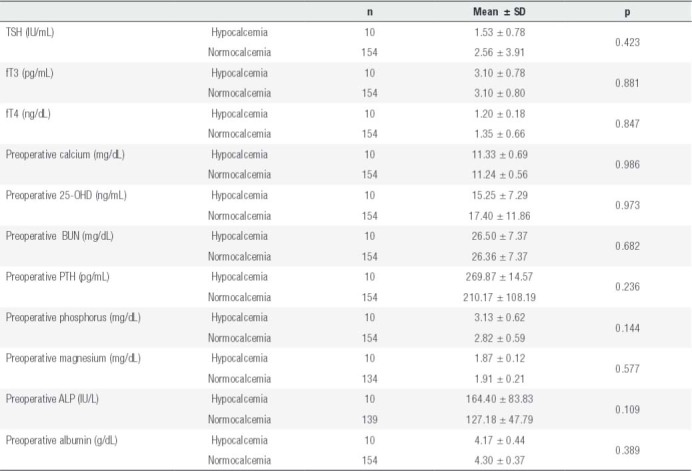
Laboratory characteristics of patients who developed permanent hypocalcemia by postoperative month 6

During the surgery, the day of the surgery, and the hospitalization period, the patients were monitored closely according to water balance and renal disorders. No kidney disorder was developed in any patients during follow-up periods.

Out of the patients who underwent parathyroidectomy for primary hyperparathyroidism (PHP), 22 (13.4%) had HBS. Out of the patients who developed HBS, 3 were male, 19 were female, and their average age was 55.7 ± 8.9 years. There was no significant difference between the patients with HBS and those without HBS with regards to age and gender (p > 0.05). Parathyroid volume (mean parathyroid volume 1.56 ± 1.26 mL) calculated with preoperative neck ultrasound was significantly higher in patients with HBS (p < 0.05). Furthermore, HBS was more common in the existence of parathyroid hyperplasia compared to parathyroid adenoma (40%, p < 0.05). While there was no increase in the risk of HBS in patients who underwent simultaneous thyroid surgery or subtotal thyroidectomy, HBS was more common in those who underwent total thyroidectomy (Table 3).

**Table 3:**
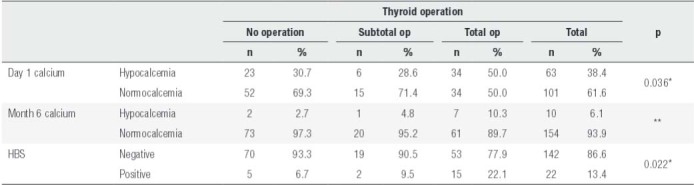
Hypocalcemia and HBS characteristics caused by having a simultaneous thyroid operation

* Values of Day 1 hypocalcemia and HBS positivity rates are observed to be significantly higher in the total thyroid operation group compared to the subtotal and no thyroid operation groups (p < 0.05).

** Statistical evaluation could not be made due to insufficient number of patients.

Additionally, preoperative PTH, ALP, and BUN levels were higher in patients who developed HBS. HBS was more common in those with osteoporosis compared to those with osteopenia (p < 0.05). History of previous neck surgery did not affect development of HBS. There was no significant difference with regards to the other biochemical markers considered (Table 4).

**Table 4:**
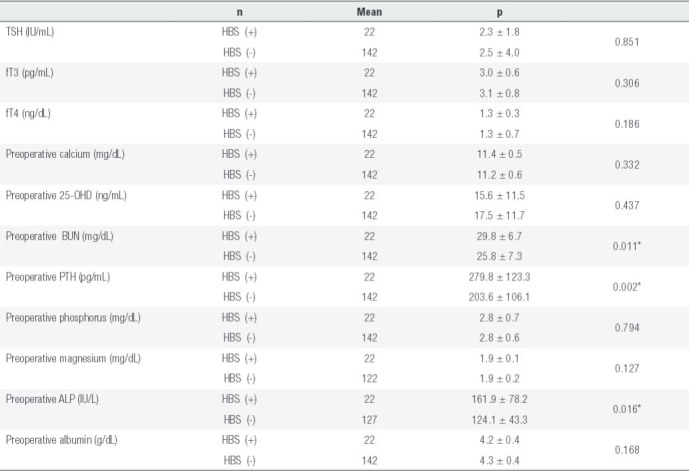
Laboratory characteristics of patients who did and did not develop HBS

HBS (+): with hungry bone syndrome; HBS (-): without hungry bone syndrome.

* p < 0.05.

## DISCUSSION

The clinical features of PHP mainly occur when excess PTH directly and indirectly affects the skeleton, kidneys, and bowels. Hypercalcemia develops with mobilization of calcium and phosphorus from the bones, increased absorption of calcium from the intestines, and renal tubular reabsorption of calcium (9).

In PHP, there is excess secretion of parathyroid hormone from one or more parathyroid glands (12). Solitary gland adenoma is the most common cause (75-85%), and while a small part of it consists of multigland adenomas, parathyroid carcinoma is rare (1%). PHP can only be cured by surgically removing the parathyroid adenoma or adenomas, and success rate in the literature is around 95-98% (13,14). In our study, the most common cause of hyperparathyroidism was created by solitary parathyroid while a double adenoma and hyperplasia of parathyroid were less present (147/164, 7/164, 10/164, respectively). There weren’t any parathyroid carcinoma cases in our study.

Postoperative hypocalcemia is one of the important and potentially dangerous complications of parathyroidectomy and has multifactorial pathogenesis. Its main mechanism is possibly insufficient parathyroid function due to slow or delayed functional recovery of the remaining parathyroid tissue after removal of the hyper-functional adenoma. Symptomatic hypocalcemia plays an important role in postponement of discharge from hospital; for this reason, some surgeons support the idea that prophylactic postoperative calcium and/or vitamin D supplementation should be given to ensure that the patient can be discharged earlier (7).

Various studies have analyzed biochemical and physical markers that may show the increased risk of developing postoperative hypocalcemia. Early identification of these risk factors in PHP patients may ensure early identification and treatment of potential postoperative hypocalcemia, and may also ensure that serious sequels are avoided. Various predictors for hypocalcemia that develops after parathyroidectomy have been investigated, but the evidence is usually controversial (5).

Mittendorf and cols. have established the percentage of postoperative hypocalcemia after parathyroidectomy in PHP patients as 42%. They have associated this with identification of all parathyroid glands and routine bilateral neck exploration. They have established that subtotal parathyroidectomy should be performed on patients who have parathyroid hyperplasia as the only factor in predicting postoperative hypocalcemia. They have not found any of the laboratory values or other clinical factors to be predictive of development of postoperative hypocalcemia (15).

Nasiri and cols. have found a correlation between preoperative calcium, PTH and ALP, and a decrease in postoperative calcium levels in a study that included 80 patients who underwent surgery for solitary parathyroid adenoma (16).

Kald and Mollerup have established that excision or incision biopsy of more than 2 parathyroid glands, concomitant parathyroidectomy and thyroid surgery, preoperative serum parathyroid hormone level higher than 25 pmol/l, or history of neck-area surgery are risk factors for development of serious postoperative hypocalcemia (3).

Crea and cols. have established that a decrease of more than 85% in intra-operative PTH is the only predictive and reliable factor in predicting serious postoperative hypocalcemia (7).

In our study, the incidence of early postoperative hypocalcemia was higher in patients who had parathyroid hyperplasia and who had osteoporosis. We have not established a correlation between preoperative PTH, ALP and calcium levels, and postoperative hypocalcemia. Patients with hyperplasia will usually require the identification, mobilization, and removal of 3 glands, with the other biopsied. These patients should be more carefully assessed with regard to hypocalcemia symptoms compared to adenomas (11).

Post-parathyroidectomy hypocalcemia can also develop because of hungry bone syndrome. Studies have shown that HBS is seen in approximately 12% of patients who undergo surgery for PHP (17). HBS ratio in patients with parathyroidectomy was 13.4% in our study. HBS is a complication of parathyroid surgery in which the correction of PHP is associated with rapid bone remineralization after surgery, causing profound and prolonged hypocalcemia which is exacerbated with suppressed PTH and associated with hypophosphatemia and hypomagnesemia (8).

Postoperative hypocalcemia develops in 90% of patients who have osteitis fibrosa cystic and serious osteoporosis. This is probably due to active storage of calcium and phosphorus in brown tumors and severely osteoporotic bones (9). Literature data related to HBS are quite limited, and unfortunately, we are not adequately familiar with the prognostic factors for development of potential HBS. The current information in the literature is based on the observations of small patient populations or a number of anecdotal individual cases (18). Various risk factors have been suggested with regard to the development of HBS. Some studies have established that PTH, ALP, and serum calcium are higher in development of HBS (8). Brasier and cols. have conducted studies on 198 patients who underwent surgery for PHP, and have found high levels of preoperative serum calcium, PTH, ALP, BUN, large parathyroid adenomas, and old age as risk factors for development of HBS (19,20).

In our study, preoperative PTH, ALP, and BUN values were higher in the patients who developed HBS. Furthermore, HBS was significantly more common in patients who had osteoporosis, who had parathyroid hyperplasia, and who underwent thyroidectomy simultaneously with parathyroidectomy.

Together with the common usage of US and scintigraphy, concomitant thyroid diseases were reported more often in the evaluation of the PHPT patients’ preoperative consultations. Hyperparathyroidism and thyroid disorder, togetherness were known in ratios up to 54% in the literature (21). Thyroidectomy (subtotal or total) was performed on 89 patients (54.4%) for various reasons (multinodular goiter, Grave’ disease) in our series. Both HBS and early hypocalcemia were investigated more often in patients with total thyroidectomy.

Serum ALP levels can serve as a marker of bone remineralization (22). Preoperative serum ALP levels reflect bone turnover situations and thus reflect the degree of osteoclastic activity and bone resorption (8). In HBS, elevated BUN can develop due to old age of patients, and the effects of hypercalcemia on renal blood flow and renal tubular function (19).

Treatment of HBS targets correction of anomalies such as hypocalcemia, hypomagnesemia and hypophosphatemia. Prevention of HBS has not been fully established. Some investigators have suggested that administration of bisphosphonates to PHP patients can prevent the development of HBS (23). Bisphosphonates have potent inhibitory effects on osteoclastic bone resorption, and are commonly used in the treatment of osteoporosis and hypercalcemia (20,24).

Vitamin D plays a critical role in the metabolism of calcium. PTH stimulates the conversion of 25-OHD into its active metabolite 1.25-dihydroxy-vitamin D3, and this may cause reduced levels of 25-OHD in PHP patients (4). It is accepted that reduced vitamin D increases the risk of postoperative hypocalcemia and development of HBS. Although vitamin D supplementation is usually recommended to normalize the 25-OHD level, there are no sufficient data showing that this can contribute to prevention of HBS (8).

Thanks to early diagnosis and suitable treatment of hyperparathyroidism before development of significant bone disease, the prevalence of post-parathyroidectomy HBS has been decreasing in recent years.

Consequently, in our study, the prevalence of postoperative hypocalcemia was significantly higher in patients who underwent parathyroidectomy for PHP, who had parathyroid hyperplasia, and who had osteoporosis. Preoperative PTH, ALP, and BUN values were significantly higher in the patients who developed HBS. Furthermore, HBS was more common in patients who had osteoporosis, who had parathyroid hyperplasia, and who underwent thyroidectomy simultaneously with parathyroidectomy. There was no significant relationship between development of early hypocalcemia and HBS, and vitamin D level. Larger, prospective, and randomized studies are needed to shed light on these controversial issues.
